# Discordant Lipid Pattern and Carotid Atherosclerotic Plaque. Importance of Remnant Cholesterol

**DOI:** 10.5935/abc.20170069

**Published:** 2017-06

**Authors:** Walter Masson, Martín Lobo, Graciela Molinero, Daniel Siniawski

**Affiliations:** 1 Hospital Italiano de Buenos Aires, Servicio de Cardiología - Argentina; 2 Consejo de Epidemiología. Sociedad Argentina de Cardiología - Argentina

**Keywords:** Atherosclerosis / complications, Plaque, Atherosclerotic, Carotid Arteries, Cholesterol, LDL, Lipoproteins, LDL, Cholesterol, VLDL

## Abstract

**Background:**

Subjects with levels of non-HDL-C 30 mg/dL above those of LDL-C (lipid discordance) or with high remnant cholesterol levels could have a greater residual cardiovascular risk.

**Objectives:**

1. To determine the prevalence of lipid discordance in a primary prevention population and analyze the clinical variables associated with it;
2. To investigate the association between lipid discordance and remnant cholesterol with the presence of carotid plaque.

**Methods:**

Primary prevention patients without diabetes or lipid-lowering therapy were included. Regardless of the LDL-C level, we define “lipid discordance” if the non-HDL-C value exceeded 30 mg/dL that of LDL-C. Remnant cholesterol was calculated as total cholesterol minus HDL-C minus LDL-C when triglycerides were < 4.0 mmol/L. Ultrasound was used to assess carotid plaque occurrence. Multiple regression logistic models were performed.

**Results:**

The study included 772 patients (mean age 52 ± 11 years, 66% women). The prevalence of lipid discordance was 34%. Male sex and body mass index were independently associated with discordant lipid pattern. The prevalence of carotid plaque was higher in subjects with lipid discordance (40.2% vs. 29.2, p = 0.002). The multivariate analysis showed that the discordant lipid pattern was associated with the greater probability of carotid plaque (OR 1.58, 95% CI 1.08-2.34, p = 0.02). Similarly, a significant association between calculated remnant cholesterol and carotid plaque was found.

**Conclusion:**

Lipid discordance and presence of a higher level of calculated remnant cholesterol are associated with subclinical atherosclerosis. Our findings could be used to improve the residual cardiovascular risk evaluation.

## Introduction

Elevations in triglyceride-rich lipoproteins are associated with an increased risk of atherosclerotic cardiovascular events even in patients with well-controlled levels of low-density lipoprotein cholesterol (LDL-C) achieved by statin regimens.^1,2^

Although LDL-C has typically been the primary target of therapy, several guidelines recognize non-HDL-C as a secondary therapeutic target.^3-6^ The US National Lipid Association (NLA) in its recent published recommendation recognizes both non-HDL-C and LDL-C as primary therapeutic targets.^7^ In this scene, the non-HDL-C targets were 30 mg/dL higher than the recommended LDL-C goals.

The non-HDL-C comprises cholesterol carried by all potentially atherogenic particles that include LDL-C, intermediate density lipoproteins, very low-density lipoproteins, remnant lipoproteins and lipoprotein(a). Additionally, in several meta-analyses, it was found that non-HDL-C correlated more closely with cardiovascular risk than LDL-C both at baseline and during therapy.^8,9^

In the same way, the “atherogenic dyslipidemia” is associated with increased cardiovascular risk. Its main findings include hypertriglyceridemia, low HDL-C levels, qualitative changes in LDL particles, accumulation of remnant lipoproteins, and postprandial hyperlipidemia.^10^ Remnant cholesterol is the cholesterol content of triglyceride-rich remnant lipoproteins, which in the fasting state comprise very low-density lipoproteins and intermediate-density lipoproteins, and, in the non-fasting state, those two lipoproteins together with chylomicron remnants. Likewise, remnant lipoproteins carry large amounts of cholesterol and share with LDL the potential to enter and get trapped in the intima of the arterial wall.^11^

On the other hand, the diagnosis of carotid atherosclerotic plaque is a surrogate objective and constitutes an independent predictor of coronary events.^12^ Our working group has previously reported a considerable prevalence of carotid plaque in patients in primary prevention.^13,14^

Given the above, we raised the possibility that subjects with non-HDL-C levels 30 mg/dL above the LDL value (lipid discordance) or with higher calculated remnant cholesterol value may show a higher prevalence of carotid atherosclerosis.

Hence, the objectives of our study were:

1. To determine the prevalence of lipid discordance in a primary prevention population and to analyze the clinical variables associated with it;
2. To investigate the association of lipid discordance and calculated remnant cholesterol with the presence of carotid plaque.

## Methods

A multicenter, descriptive, cross-sectional study was performed on consecutive samples obtained in the cardiovascular prevention outpatient clinics of six cardiology centers in the Autonomous City of Buenos Aires. Primary prevention subjects were included. Exclusion criteria were:

1. previous cardiovascular disease;
2. history of diabetes mellitus; and
3. prior hypolipidemic therapy.

Colorimetric and turbidimetric assays were used to measure non-fasting plasma levels of triglycerides, HDL-C and total cholesterol. The Friedewald equation was used to calculate LDL-C. Remnant cholesterol was calculated as total cholesterol minus HDL-C minus LDL-C when triglycerides were < 4.0 mmol/L.

Regardless of the LDL-C level, “lipid discordance” was defined as a non-HDL-C level exceeding 30 mg/dL that of LDL-C.

Carotid atherosclerotic plaque was recorded when atherosclerotic plaque was found in the carotid arteries on noninvasive, 2D-mode ultrasound images. Presence of plaque was defined as:

- abnormal wall thickness (defined as intima-media thickness >1.5 mm);
- abnormal structure (protrusion towards the lumen, loss of alignment with the adjacent wall); and
- abnormal wall echogenicity.

Three cardiovascular risk scores were calculated:

1. The Framingham Score for coronary events using the third National Cholesterol Education Program (NCEP) expert panel report on elevated blood cholesterol detection, assessment and treatment in adults (Adult Treatment Panel III - ATP III),^15^ defining low, moderate and high risk as values < 10%, between 10% and 19%, and ≥ 20%, respectively;
2. The new score used by the last 2013 ACC/AHA Guidelines for cholesterol management;^16^
3. The European SCORE for fatal events, using the specific score corresponding to low risk countries.^17^ The choice of this score was arbitrary, based on the fact that most Argentine immigrant population comes from those countries. Risks < 1%, between 1% and 4.9%, 5% and 9.9% or ≥ 10% were classified as low, moderate, high or very high, respectively.

### Statistical analysis

Variable normality was explored analyzing the mean, standard deviation, median, skewness, kurtosis and histogram, and using the Shapiro-Wilk test. Continuous data were compared between groups using the unpaired *t* test for normal distribution or the Mann-Whitney-Wilcoxon test for non-normal distribution. The analysis of categorical data was performed using the chi-square test. The correlation between LDL-C and non-HDL-C was performed with the Pearson test.

A multiple regression logistic model was performed to identify independent characteristics associated with discordant lipid pattern, including all variables with p < 0.05 in the univariate analysis or those considered clinically relevant (age and smoking). Similarly, another multiple regression logistic model was performed to explore the association between the discordant lipid pattern and the presence of carotid plaque, including all variables with p <0.05 in the univariate analysis or those considered clinically relevant (sex). Finally, a third multivariate model was performed to analyze the association between highest and lowest quartiles of calculated remnant cholesterol and the presence of carotid plaque, adjusting for age, sex, body mass index, smoking and antihypertensive medication.

Continuous variables were expressed as mean (standard deviation) if the distribution was normal and as median (interquartile range) if the distribution was abnormal. Categorical variables were expressed as percentages. A two-tailed p value < 0.01 was considered as statistically significant. STATA 11.1 and 3.1 EPIDAT software packages were used for statistical analysis.

Sample size calculation: Looking to have a power of 80% and an alpha error of 0.05 to detect an absolute difference equal to or greater than 7% in the prevalence of carotid plaque among subjects with or without lipid discordance, we estimate that it would be necessary a sample of 513 subjects. Assuming a loss of 15%, the number was 604 patients.

### Ethics considerations

The study was conducted following the recommendations in medical research suggested by the Declaration of Helsinki, Guidelines for Good Clinical Practice and valid ethical regulations. The ethical issues have been evaluated and approved by the Area of Investigation of the Argentine Society of Cardiology.

## Results

A total of 772 patients (mean age 52 ± 11 years, 66% women) were included in the study. Average body mass index was 26.9 ± 4.5 and mean cholesterol, LDL-C and HDL-C values were 219 ± 45 mg/dL, 142 ± 43 mg/dL and 50 ± 14 mg/dL, respectively. The median triglyceride level was 117 mg/dL (80-173). Thirty-six percent of patients were receiving antihypertensive treatment, and 20.5% were active smokers.

**Figure 1:**
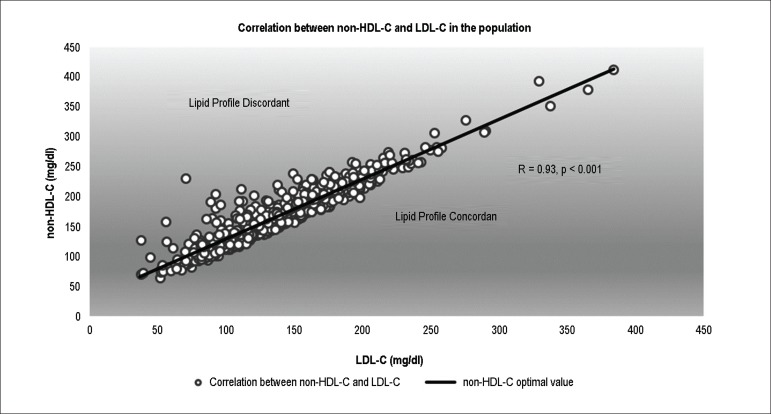
Relationship between LDL-C and non-HDL-C in the population. The black line represents the value of non-HDL-C (30 mg/dL above) associated with each value of LDL-C.

Thirty-four percent of the population showed lipid discordance (non-HDL-C > LDL-C+30 mg/dL). Relationship between LDL-C and non-HDL-C in the population is shown in Figure 1.

Patients with lipid discordance showed a higher proportion of men and subjects on anti-hypertensive medication, and higher body mass index compared with subjects with lipid concordance. Moreover, a smaller proportion of low-risk individuals according to different cardiovascular scores was observed in the group with lipid discordance. The characteristics of the population according to the lipid pattern are shown in Table 1. Also, lipid values according to the lipid pattern are displayed in Table 2.

**Table 1:** Association between different non-lipid risk factors and lipid pattern

	Concordant n = 510	Discordant n = 262	p
**Continuous variables, mean (SD)**			
Age, years	52.8 (10.8)	51.6 (11.3)	0.15
Systolic blood pressure, mm Hg	127.5 (15.3)	129.1 (14.5)	0.16
Body mass index, kg/m^2^	26.0 (4.2)	28.6 (4.6)	< 0.001
**Categorical variables, %**			
Male sex	39.0	54.6	<0.001
Anti-hypertensive medication	33.1	42.8	0.01
Smoking	20.2	21.0	0.79
Family history of early cardiovascular disease	26.5	26.4	0.98
**Framingham score (ATP III)**			
Low risk	80.6	69.4	0.001
Intermediate risk	13.9	24.5	
High risk	5.5	6.1	
**New Score (ACC/AHA 2013)**			
< 5%	55.1	40.8	0.001
5-7.5%	10.8	16.8	
> 7.5%	34.1	42.4	
**European SCORE**			
Low risk	53.3	46.2	0.04
Intermediate risk	36.9	47.0	
High/Very high risk	9.8	6.8	

SD: standard deviation.

**Table 2:** Lipid values according to the lipid pattern

Variable (mg/dL)	Concordant n = 510	Discordant n = 262	p
Total Cholesterol, mean (SD)	213.3 (44.3)	232.2 (44.9)	< 0.001
LDL-C, mean (SD)	141.5 (41.8)	142.8 (44.1)	0.58
Non-HDL-C, mean (SD)	160.0 (42.9)	188.4 (43.1)	< 0.001
HDL-C, mean (SD)	53.3 (14.7)	43.8 (11.3)	< 0.001
Triglycerides, median (interquartile range)	89.0 (72.0-116.0)	199.0 (172.0-252.0)	< 0.001
Calculated remnant cholesterol, mean (SD)	18.5 (5.8)	45.1 (16.3)	< 0.001

SD: standard deviation.

In the multivariate analysis, male sex (OR: 1.50; 95% CI: 1.07-2.11, p = 0.02) and body mass index (OR: 1.12; 95% CI: 1.08-1.17, p < 0.001) were independently associated with greater probability of being a discordant lipid pattern.

Table 3 displays the characteristics of the population (non-lipid risk factors) according to the presence or absence of carotid plaque. The prevalence of carotid plaque was significantly higher in subjects with lipid discordance (40.2% vs. 29.2, p = 0.002). In univariate analysis, a significant association between discordant lipid pattern and the presence of carotid plaque was observed (OR: 1.61; 95% CI: 1.17-2.19, p = 0.003). In the same way, the multivariate analysis showed that the discordant lipid pattern was independently associated with greater probability of exhibiting carotid plaque (OR: 1.58; 95% CI: 1.08-2.34, p=0.02). This finding occurred after adjusting for age, sex, body mass index, systolic blood pressure, antihypertensive treatment, active smoking and family history of early coronary disease.

**Table 3:** Association between non-lipid risk factors and carotid plaque

	Without plaque n = 517	With plaque n = 254	p
Continuous variables, mean (SD)			
Age, years	49.8 (11.3)	57.7 (11.3)	< 0.001
Systolic blood pressure, mm Hg	125.2 (13.8)	133.8 (15.9)	< 0.001
Body mass index, kg/m^2^	26.3 (4.5)	27.9 (4.4)	< 0.001
Categorical variables, %			
Male sex	42.0	48.9	0.07
Anti-hypertensive medication	28.4	52.8	<0.001
Smoking	15.1	31.1	<0.001
Family history of early cardiovascular disease	23.8	32.0	0.016

SD: standard deviation.

**Figure 2:**
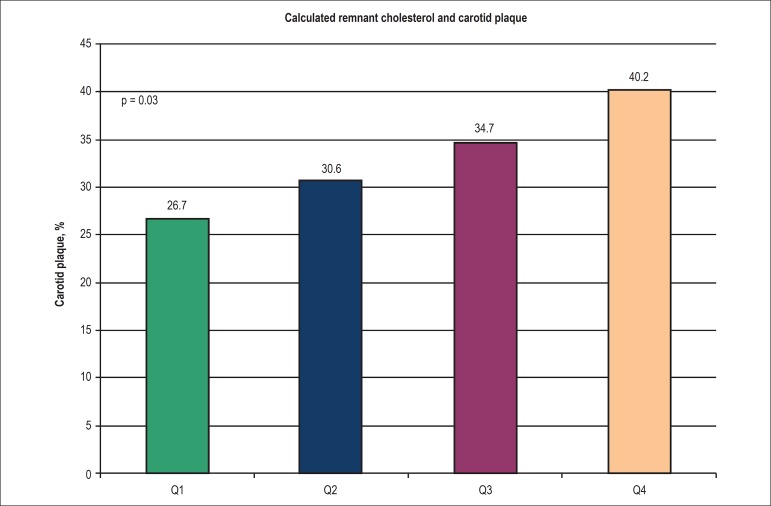
Association between quartiles of calculated remnant cholesterol and the presence of carotid plaque. Q: Quartile.

Similarly, a significant association between calculated remnant cholesterol and the presence of carotid plaque was found in the univariate analysis (upper vs. lower quartile: OR: 1.82; 95% CI: 1.19-2.79, p = 0.006). This association remained after adjusting for other risk factors (upper vs. lower quartile: OR: 1.84; 95% CI: 1.11-3.05, p = 0.02). Figure 2 shows the association between quartiles of calculated remnant cholesterol and the presence of carotid plaque.

## Discussion

Discordance analysis is an analytical technique in which biologically linked variables are analyzed by groups of concordance or discordance between their relative distributions.^18^ In our work, we have defined "lipid discordance" arbitrarily but in an original way. For each patient, we categorize the lipid pattern as discordant if the non-HDL-C level exceeded 30 mg/dL that of LDL-C. Thus, the clinical value of this analysis is more closely related to the number of atherogenic particles than to the total mass of cholesterol.

In our study, male patients or subjects with a higher body mass index were more likely to show lipid discordance. Coinciding with our findings, in a study that analyzed a population of Barcelona, the prevalence of atherogenic dyslipidemia was higher in men compared to women (using the HDL-C cutoff recommended by the European guidelines).^19^ Also, a study conducted in primary health care users of Portugal showed a higher prevalence of hypertriglyceridemia and low HDL-C levels in males.^20^ In another study, Williams et al.^21^ demonstrated that non-diabetic men showed higher levels of apolipoprotein B, non-HDL-C and small LDL particles than women without diabetes. On the other hand, the association between overweight or obesity and high non-HDL-C levels was widely demonstrated.^22,23^

Our analysis showed an association between discordant lipid pattern and higher prevalence of carotid plaque. Similarly, Holewijn et al.^23^ showed that subjects with high non-HDL-C levels had a lower ankle-brachial index, increased mean intima-media thickness and more atherosclerotic plaques than patients with low non-HDL-C levels. Moreover, high non-HDL-C/low LDL-C discordance was associated with higher coronary artery calcium score measured by computed tomography.^24^ Consequently, several factors arise to explain the association between discordant lipid pattern and higher prevalence of subclinical atherosclerosis. First, some triglyceride-rich lipoprotein remnants enter the arterial wall similarly to LDL-C, contributing to the initiation and progression of atherosclerosis. Second, non-HDL-C correlates more closely with the total burden of all atherogenic particles. Finally, elevated levels of triglycerides and very low-density lipoprotein-C could reflect the hepatic overproduction of atherogenic and dense particles characterized by a slower clearance from the circulation.

Another finding of our study was the association between calculated remnant cholesterol and the presence of carotid plaque. This association remained even after adjusting for non-lipid risk factors.

Elevated remnant cholesterol is associated with ischemic heart disease.^25^ Similarly, increased concentrations of both calculated and measured remnant cholesterol were associated with increased all-cause mortality in patients with ischemic heart disease.^26^ The main explanation for a causal effect of elevated remnant cholesterol on ischemic heart disease risk could be that remnants enter and get trapped in the intima of the arterial wall.^27^ Even more, remnants may not need to be oxidized to be taken up by macrophages to cause foam cell formation and atherosclerosis.^28^ Therefore, the findings of our research are consistent with pathophysiological and clinical data previously reported.

Our findings, regarding that patients with discordant lipid patterns and higher remnant cholesterol levels are associated with atherosclerotic plaque, highlight the role of non-HDL-C in clinical practice. In the real world, patients with very high cardiovascular risk have a significant prevalence of atherogenic dyslipidemia despite having achieved LDL-C goals.^29^

However, the recommendations of the guidelines are confusing and not always consistent.^30^ The 2013 ACC/AHA guidelines for cholesterol management do not consider HDL-C and triglycerides in cardiovascular prevention. However, the NLA emphasizes the relevance of atherogenic dyslipidemia and the Canadian guidelines introduced non-HDL-C and apolipoprotein B as alternative targets. The International Atherosclerosis Society and National Institute for Health and Care Excellence (NICE) guidelines promote the importance of non-HDL-C. The European guidelines highlight HDL-C and triglycerides, but with the limitation that the main evidence comes from sub-analysis of clinical studies.

Our study has some limitations. First, as in any cross-sectional study, the possibility of bias (mainly selection bias) potentially influencing the results cannot be ruled out. We believe a selection bias may exist in our sampling, as patients attending the cardiovascular prevention clinic do not necessarily represent the general population. Second, we did not measure remnant lipoprotein cholesterol directly. However, the measurement of calculated remnants has been used in several previous studies. Finally, in our study, carotid plaque was defined according to the Atherosclerosis Risk in Communities study criteria. Changing the definition of plaque could modify our results.

## Conclusion

In our analysis, the lipid discordance and the presence of a higher level of calculated remnant cholesterol are associated with subclinical atherosclerosis. Our findings expand the strategies in primary prevention to evaluate the residual cardiovascular risk.
